# Application of a Rapid and Simple Technological Process to Increase Levels and Bioccessibility of Free Phenolic Compounds in Annurca Apple Nutraceutical Product

**DOI:** 10.3390/foods11101453

**Published:** 2022-05-17

**Authors:** Maria Maisto, Elisabetta Schiano, Ettore Novellino, Vincenzo Piccolo, Fortuna Iannuzzo, Emanuela Salviati, Vincenzo Summa, Giuseppe Annunziata, Gian Carlo Tenore

**Affiliations:** 1 Department of Pharmacy, University of Naples Federico II, via Domenico Montesano 59, 80131 Naples, Italy; maria.maisto@unina.it (M.M.); elisabetta.schiano@unina.it (E.S.); vincenzo.piccolo3@unina.it (V.P.); fortuna.iannuzzo@unina.it (F.I.); Vincenzo.summa@unina.it (V.S.); giancarlo.tenore@unina.it (G.C.T.); 2 Faculty of Medicine, University Cattolica, Largo Agostino Gemelli, 00168 Rome, Italy; Ettorenovellino09@gmail.com; 3 Department of Pharmacy, School of Pharmacy, University of Salerno, 84084 Fisciano, Italy; Esalviati@unisa.it

**Keywords:** annurca apple polyphenols, polyphenolic bioaccesibility, insoluble bound polyphenols, technological process, acid treatment, pectin depolymerization

## Abstract

Insoluble bound polyphenols (ISBP) are polyphenolic compounds linked to the food matrix with different interactions limiting both their water extractability and consequent bioaccessibility. The health-promoting potential of polyphenols is historically known and well-demonstrated; specifically, Annurca apple polyphenols were studied both in vitro and in vivo for their effect in controlling cholesterol plasma levels. The aim of the study was the preparation of nutraceutical products based on Annurca apple polyphenolic fraction through the application of a technological process (acid treatment) able to release the ISBP from Annurca apple food matrix and increase polyphenol bioaccessibility. Lyophilized annurca apple (LAA) underwent acid treatment (ATLAA), and differences in released polyphenol levels were analysed by DAD-HPLC. Free-polyphenol levels in samples treated under acid conditions were higher than in untreated ones; in particular, for oligomeric flavan-3-ols (+168% procyanidin B2, +42.97% procyanidin B1 and B2, +156.99% procyanidin C1), catechin (+512.20%), and gallic acid (+707.77%). Furthermore, ATLAA underwent an in vitro gastrointestinal digestion to evaluate the bioaccessibility of contained polyphenols, in comparison to the untreated Annurca apple. The bioaccessibility study indicates a valuable preservation of polyphenolic fraction compared to the control.

## 1. Introduction

Due to their peculiar chemistry, polyphenols are linked to insoluble constituents of food matrix (manly comprised of carbohydrate polymers like cellulose, hemicellulose, pectin, and lignin [1]) and specifically to the plant cell wall components (PCW) by different kinds of interactions, including covalent linkage, hydrogen bonding, and hydrophobic interactions [2]. Such polyphenols, named insoluble bound polyphenols (ISBP), have a very low water solubility and, thus, are poorly extractable from food matrices [3,4,5,6,7,8]. More specifically, conventional extraction process based on the use of hydroalcoholic solvents are not able to extract efficiently these polyphenols from the food matrix [8,9,10], this extraction, however, can be achieved following chemical or enzymatic hydrolyses.

Various chemical and biological methods have been developed to promote the release of ISBP from food matrices. The biological methods are based on the use of carbohydrate-hydrolyzing enzymes, such as pectinase, cellulase, hemicellulase, and glucanase, able to release polyphenols complexed with the cell wall [11,12,13]. On the other hand, the rate of free phenolic compounds may be chemically increased by using acid and/or alkaline treatments. Alkaline and acid hydrolysis have different effects in releasing phenolics from the same food matrix [14,15,16], due to both kind and proportion of covalent bonds between phenolics and food matrix [17].

Li et al. investigated the effects of acid and/or basic hydrolysis in sequence or alone on the rate of free phenolic compounds in apple pomace. They found that, the most effective treatment in terms of release of total phenolic content (TPC) was the use of sequential acid-basic hydrolysis. Therefore, they reported that the single acid treatment (2 M HCl) was able to increase selectively the amount of procyanidin B2 in samples treated in acid conditions [17]. Moreover, acid hydrolysis is mainly used for apple pectin depolymerization [18], resulting in release of polyphenols complexed into the tridimensional pectin structure. Pectin is the main component of fruit polysaccharides, belonging to a family of complex variable polysaccharides extracted from the primary PCW of higher plants. Chemically, it is a linear polymer of D-α-(1→4) and hydrogalacturonic acid [19].

Previous studies reported the key role played by polyphenolic fraction of Annurca apple (the only apple cultivar native to Southern Italy, listed as a Protected Geographical Indication (PGI) product from the European Council [Commission Regulation 0(EC) No. 417/2006]) on prevention and treatment of metabolic syndrome, both in vitro and in vivo experiments. This evidence was confirmed in humans with two randomized clinical trials, conducted on heathy subjects administered with both Annurca apple fruit and a nutraceutical formulation based on polyphenolic aqueous extract from Annurca apple [20,21]. In this sense, ISBP of Annurca apple fruit may be a considerable source of additional potential bioactive molecules, which heathy activity may be affected by their linkage to the pectin by three types of bounds: hydrophobic interaction, hydrogen bonds and ester bond [13].

The evaluation of polyphenolic bioaccessibility is a key factor in assessing their significance in human health. To exert their biological activities in humans, polyphenols have to be available to some extent to the target tissue. It is well accepted, however, that polyphenols are characterized by a very low bioaccessibility and bioavailability, not only due to their low water solubility [22], but also to several food matrix-related factors, such as the linkage of polyphenols with the PCW polysaccharides [22,23]. Moreover, recent studies have demonstrated that, chemical and/or chemical-physical treatment of food matrixes may lead to either a prompt release of polyphenols, or remodeling of fiber structure, which allows accessibility of polyphenols-carbohydrate linkages to hydrolytic enzymes at intestinal level. Zhang et al. have found that pumpkin carotenoids bioaccessibility was increased after the deconstruction of food matrix by microwave treatments [24]. On the other hand, Rojas et al., have obtained a remarkable increase of polyphenol bioaccessibility after acidic hydrolysis of food polysaccharides food matrix of raspberry residues [25].

In the light of what is stated above, the main aim of the present work is the preparation of an innovative product by application of food grade, rapid, simple acid treatment protocol aimed to increase the rate of free phenolic compounds in lyophilized Annurca apple (ATLAA, Acid Treated Lyophilized Annurca Apple). In the second instance, we evaluated the intestinal bioaccessibility of ATLAA in comparison to that of lyophilized not treated Annurca apple (LAA). For the analysis of polyphenolic profile, an accurate, reproducible, and precise HPLC-DAD method was validated.

## 2. Materials and Methods

### 2.1. Acid Treatment (AT)

The method used for acid treatment was previously described by [25] and applied with slight modifications. Annurca (Malus pumila Miller cv Annurca) apple fruits (each, about 100 g) were collected in Valle di Maddaloni (Caserta, Italy) in October when fruits had just been harvested (green peel). The fruits were reddened, following the typical treatment for about 30 days, and then analyzed. After this time, apples were washed and cut in slides to be lyophilized. An amount of 1 g of whole Annurca apple lyophilized (peel and pulp) was suspended in 20 mL of deionized water, vortexed for 2 min, and brought to pH = 2.6 by the addition of HCl 0.1 N. The obtained mixture was left in agitation at room temperature for 2 h on an orbital shaker (Sko-DXL, Argolab, Carpy, Italy) at 300 rpm. After this time, the obtained mixture was adjusted to pH = 7 by the addition NaOH 0.1 N, and further lyophilized; the powder obtained (Acid Treated Lyophilized Annurca apple, ATLAA) was stored at −4 °C until the analysis. The control (CTR) was prepared with the same procedure, with the replacement of the HCL 0.1 N with the same volume of deionized water.

### 2.2. Polyphenolic Extraction

A volume of 10 mL of 80% methanol, 1% formic acid was added to 1.5 of lyophilized samples (ATLAA) and control; the mixture was homogenized for 5 min by ultraturrax (T25-digital, IKA, Staufen im Breisgau, Berlin, Germany) and shaken on an orbital shaker (Sko-DXL, Argolab, Carpy, Italy) at 300 rpm for 15 min. Then, the samples were placed in an ultrasonic bath for 10 min, and centrifuged at 6000 rpm for 10 min. The supernatants were collected and stored away from light at 4 °C. The pellets obtained were re-extracted with the same procedure using 5 mL of the same extraction solvents. Finally, the extracts obtained were filtered on syringe filter (0.45 mm in RC, Phenomenex, Torrance, CA, USA) and stored at −20 °C until the analysis.

### 2.3. HPLC-DAD Quantitative Polyphenols Determination

Analyses were run on a Jasco ExtremaLC-4000 system (Jasco Inc., Easton, MD, USA) provided with photodiodearray detector (DAD). The column selected was a Kinetex^®^C18 column (250 mm × 4.6 mm, 5 μm; Phenomenex, Torrance, CA, USA). The analyses were performed at a flow rate of 1 mL/min, with solvent A (2% formic acid) and solvent B (0.5% formic acid in acetonitrile and water 50:50, *v*/*v*). After a 5 min hold at 10% solvent B, elution was performed according to the following conditions: from 10% (B) to 55% (B) in 50 min and to 95% (B) in 10 min, followed by 5 min of maintenance. Flavonols, procyanidins, dihydrochalcones, flavanols, and hydroxycinnamic acids were monitored at 280 nm and flavan-3-ols at 360 nm (Supplementary Materials, Figure S1 and Table S1). For quantitative analysis, standard curves for each polyphenol standard were prepared over a concentration range of 0.1–1.0 μg/μL with six different concentration levels and duplicate injections at each level. The identity of polyphenols was confirmed by comparison of retention time of analytical standard and by internal standard analysis (Supplementary Materials, Figure S2). The percentage variation (PV) respects the control was calculated for each polyphenol according to the following formula:

$$\%\mathrm{PV}\;=\frac{\left[\mathrm{polyphenolic}\;\mathrm{compound}\;\left(\mathrm{mg}/g\right)\;\mathrm{in}\;\mathrm{ATALL}\right]-\left[\left(\mathrm{polyphenolic}\;\mathrm{compound}\;\left(\mathrm{mg}/g\right)\;\mathrm{CTR}\right)\right]}{\left[\left(\mathrm{polyphenolic}\;\mathrm{compound}\;\left(\mathrm{mg}/g\right)\;\mathrm{in}\;\mathrm{CTR}\right)\right]}\times 100.\;$$

### 2.4. Method Validation

#### 2.4.1. Linearity and Sensitivity

Standard compounds of gallic acid, procyanidin B1, procyanidin B3, catechin, chlorogenic acid, procyanidin B2, epicatechin, rutin, phlorizin, and quercetin were used to develop and validate the method. Stock solutions of all the standards were prepared at a concentration of 1.0 μg μL^−1^, using HPLC grade methanol as solvent. Six different concentrations (0.0005, 0.001, 0.005, 0.01, 0.05, and 0.1 mg/mL) of each standard were prepared from standard stock solutions and analyzed on the HPLC in duplicate. A six-point calibration curve was drawn by plotting peak area versus concentration of respective standards. The concentration range was 0.0005–0.1 mg/mL for all analytes, with a correlation coefficient of *r*^2^ = 0.999 for all analytes expect for procyanidin C1 and catechin. The limits of detection (LODs) and limits of quantification (LOQs) were determined to evaluate sensitivity of the method. Determination of the signal-to-noise ratio is performed by comparing measured signals from sample with known low concentrations of analyte with those of blank samples and establishing the minimum concentration at which the analyte can be reliably detected as previously described [26]. LODs is defined as the lowest detectable concentration of analyst that analytical system can reliably differentiate from the background level (S (signal of compound)/N (signal of noise)) = 3, while LOQ is defined as the lowest quantifiable level of analyst that can be measured with standard level of confidence and it is typically calculated using (S/N) = 10 [26]. In the following table are reported the value of LOQs, LODs and linearity related to each analyte identified and quantified in the extract.

#### 2.4.2. Precision and Accuracy

Accuracy (% bias) was determined by an intraday and inter-day analysis of calibration standards. Each analyte was injected three times per day (intra-day) and one time for three consecutive days (inter-day). Precision (%CV, coefficient of variation %) was determined by an intraday and inter-day analysis of calibration standards. Each analyte was injected three times per day (intra-day) and one time for three consecutive days (inter-day).

### 2.5. In Vitro Gastrointestinal Digestion

An amount of 2.5 g of freeze-dried sample was subjected to simulated in vitro gastrointestinal digestion following a previously described method [27], with few modifications. Lyophilized samples were suspended in 1.75 mL of simulated salivary fluid. After one min of stirring, 0.25 mL of α-amylase solution (in simulated salivary fluid, 75 U/mL) 12.5 µL of 0.3 M calcium chloride, and 488 µL of water were added. Then, the pH of the mixture was adjusted to seven with HCl 1 M and the solution was incubated in a shaker bath (100 cycles/min) at 37 °C for 2 min. The gastric phase was performed by adding to the oral bolus, 3.75 mL of simulated gastric fluid, 0.8 mL of pepsin solution (suspended in simulated gastric fluid, 2000 U/mL), and 2.5 µL of 0.3 M calcium chloride. The pH of the solution was adjusted to three with HCl 1 M, the volume was filled up to 10 mL with distilled water and the samples was incubated in a shaker bath (100 cycles/min) at 37 °C for 2 h. Then, in order to simulate the duodenal conditions, gastric digested was added with 5.5 mL of simulated intestinal fluid, 2.5 mL pancreatin solution (suspended in simulated intestinal fluid, 100 U/mL of trypsin activity), 20 µL of 0.3 M calcium chloride, and 1.25 mL bile salt solution (65 mg/mL); the mixture was thoroughly amalgamated and the pH of the solution was reported to 7 with NaOH 1 M. Then, the volume was brought to 20 mL with water, the mixture was incubated in a shaker bath (100 cycles/min) at 37 °C for 2 h and then centrifuged at 4900× *g* at 37 °C for 10 min. The supernatant phase (after duodenal phase) was collected for the analysis.

### 2.6. Statistics

Unless otherwise stated, all the experimental results were expressed as the mean ± standard deviation (SD) of three determinations. Statistical analysis of data was performed by the student’s *t*-test. *p* values less than 0.05 were regarded as significant.

## 3. Results

### 3.1. ATLAA Polyphenolic Composition

Qualitative analysis does not indicate a variation of the polyphenolic composition of ATLAA compared to the CTR. On the other hand, a relevant change in terms of total amount of extractable polyphenolic compounds was detected. As shows in Table 1, for all the class of polyphenols, a marked increase was evaluated compared to the control. Particularly, for flavan-3-ols class a relevant polyphenolic release was observed. Specifically, procyanidin B2 and C1 concentrations were increased of 168.26% and 156.99% respectively, while for the monomeric species (catechin and epicatechin) the improvements were of 77.47% and 512.49%, respectively. For the class of flavonols the more relevant and significative (*p* < 0.05) releasing was observed for Apigenin-7-O-glucoside (with an increase of 29.04% vs. ctr), for the other compounds of this class a not significative increase was observed. A 90% increase was observed for phlorizin and 707% for gallic acid. Overall, the increase of free phenolic compounds in formulated product was of 49.51% compared to the control.

### 3.2. ATLAA and LLA Polyphenolic Bioaccessibility

To evaluate the polyphenol bioaccessibility of the formulated products, a protocol of in vitro gastrointestinal digestion was performed on ATLAA and LAA as described above. Interestingly, from a comparative analysis of the polyphenolic composition between ATLAA and LAA digested fractions, a remarkable increase of free and bioaccessible phenolic compounds was detected the formulated product (Table 2). The overall increase of total polyphenols detected in ATLAA was around 17.36% compared to LAA digested fraction; in particular, the concentration of the flavonol esperidin increase of 128.29% compared to the control. Generally, for flavonols, a relevant preservation after in vitro gastrointestinal digestion was observed. For most molecules belonging to this class, including kampherol-3-O-glucoside, apigenin-7-O-glucoside, and naringenin, remarkable conservation was achieved (51.38, 29.21, and 96.45% higher than LAA digested fraction, respectively). Regarding dimeric (procyanidins B1, B2 and B3) and trimeric (procyanidins C1) flavan-3-ols, we evaluated their considerable preservation after the in vitro digestion that results in increases of 30%, 22%, and 18.42%, respectively, compared to the control. Moreover, epicatechin was completely degraded by enzymatic activity.

### 3.3. Precision and Accuracy

The intra-day and inter-day accuracy (% bias) and precision (% CV) were determined at the concentrations of 0.1, 0.05 and 0.01 mg/mL for all the standards tested (Table 3). As expected, the higher % CV was measured at lower concentration tested for most of the molecules studied. As reported in Table 3, polyphenols detected more precisely are the favolnols, with a lower CV% values and with low concentration dependent variation of CV% value for each molecule evaluated (rutin and quercetin). On the other hand, the polyphenolic class characterized by higher CV% are flavan-3-ols; indeed, for the procyanidin B2, for example, the higher CV% inter-day with values of CV% 10.747 (at 0.01 mg/mL) was described, while the higher CV% inter-day was evaluated for epicatechin (0.01 mg/mL). The same was observed for the accuracy (Table 4), where quercetin and rutin were detected with lower and homogenous bias%. We have found that rutin detection was described by lower intraday and inter-day bias% detected, with low collection of data variability between the different concentration levels tested. Among tested molecules, a lower accurate detection has been observed, with bias% ranging from 1.88 to 19.22 (intra-day) and from 1.92 to 19.40 (inter-day). Generally, we have found that % CV values for all the analytes studied ranged from 0.16 to 8.86% and from 0.122 to 10.74% for inter-day and intraday precision, respectively; the % bias ranged from −2.96 to 4.32 for the estimation of intraday accuracy and from −2.98 to 19.40% for the evaluation of inter-day accuracy.

### 3.4. Linearity and Sensitivity

Linearity studies were conducted by the preparation of calibration curves on a wide range of analytical standard concentrations (seven dilutions ranging from 0.001 to 0.1 mg/mL). All the analyses were conducted in triplicate, the ratio of standards concretions versus peak area ratio (analyte peak area/internal standard peak area) was plotted with a correlation coefficient of R 2 0.999. the sensitivity of the analytical method was examined for each one molecule studied resulting il LOD e LOQ values reported in Table 5.

## 4. Discussion

Our results indicate a marked increase of free phenolic compounds in ATLAA product obtained after acid treatment compared to the control. Although the acid and/or alkaline hydrolysis are chemical treatments largely used in transformation and production of food products and nutraceuticals, in this study, the acid treatment was used as bifunctional strategy. On one hand, it is a useful tool to increase the rate of free phenolic compounds in nutraceutical product; on the other hand, it is able to cause remodeling and depolymerization of Annurca apple food matrix that, potentially, results in a higher intestinal polyphenolic bioaccessibility. The increase of free phenolic compounds in ATLAA was significantly detected only for the flavan-3-ols (e.g., catechin +512%, procyanidin B2 +168.20% and procyanidin C1 +156.99%) and phenolic acid (gallic acid +707.2%). A not significant variation was observed flavonols (rutin, quercetin and kamperhol-3-O-rhamnoside). This evidence may be related to the different type of interactions between the flavan-3-ols, phenolic acid, and flavonols with the food matrix. In apple, the food matrix is manly represented by pectin. Chemically, pectin is a family of complex variable polysaccharides extracted from the primary cell wall of higher plants.

The acid treatment (for 1.5 h, at pH 2.8) changes the charge state of pectin (predominantly the not ionized form, pka = 3) [12]. In this condition the alcoholic functions of pectin are in a protonated form, so not able to form an ester link to the carboxylic group of phenolic acid (e.g., gallic acid +707.2%). Regarding oligomeric flavan-3-ols, the chemical mechanism proposed to explain the relevant release obtained is different (e.g., catechin +512%, procyanidin B2 +168.20% and procyanidin C1 +156.99%). The literature indicates that oligomeric flavan-3-ols, are linked to the food matrix constituents by hydrophobic interaction and hydrogen bound [12,28]. These types of interactions increase exponentially with the molecular weight of procyanidins (grade of polymerization), probably due to the increase in the molecular size of polymeric flavan-3-ols (procyanidins), resulting in more ortho-phenolic groups (able to form hydrogen bonds) and aryl rings (able to form hydrophobic interactions) per molecule with the polysaccharides of food matrix [12,28]. In this sense, procyanidins B1, B2, and B3 (dimeric) are more strongly linked to the food matrix than catechins, and less than procyanidin C1 (trimeric). In addition, in acid condition, apple pectin could undergo acid hydrolysis and β-elimination reactions, which results in the demethoxylation and depolymerization of homogalacturonan [29,30,31]. This remodeling of pectin structure, that influences its tridimensional structure and folding, causing a decreasing of hydrophobic interaction, results in a marked release of procyanidins. Among the monitored polyphenolic compounds of Annurca apple, procyanidins are the molecules with higher molecular weight, for this reason, a not considerable release was observed for molecules with lower molecular weight not strongly involved in hydrophobic interactions (flavonols). However, this does not explain, despite its low molecular weight, the relevant catechin increase in ATLAA (512.49%). This event may be explained, considering the type of linkage between catechin and pectin. Monomeric flavan-3-ols are bond to pectin also through aryl-ether linkages, that is rapidly cleavage in acidic condition [1].

Evaluation of intestinal bioaccessibility has a key role in the formulation of innovative nutraceutical products due to the very low intestinal bioaccessibility of polyphenols. The release of dietary polyphenols from food matrix is mediated by the combination of intestinal enzyme activities (small intestine) and fermentation by gut microbiota (large intestine). In this product, the acid treatment used may be consider as a potential pre-digestion, with a preliminary release of trapped polyphenols by cleavage of linkage pectin-polyphenols, and by pectin depolymerization, that combined in vivo with the activity of both intestinal enzymes and gut microbiota, may results in higher polyphenols bioaccessibility [32]. In this context, our results are perfectly in line with these considerations, with an overall increase of polyphenols bioaccessible fraction of 17.36% higher than the LLA. Specifically, the flavan-3-ols are the class more preserved after in vitro digestion. This may be due to the remodeling of food matrix caused by acid treatment that makes the pectin and in general food matrix more accessible for the digestive enzymes, resulting in release of the polyphenols still linked to the polysaccharide matrix.

## 5. Conclusions

Based on such promising results ATLAA may be considered as a prototype of nutraceutical product based on Annurca apple polyphenols. The potential of this product, maybe due to the rate of free phenolic compounds and the relevant increase of bioaccessibility. Undoubtedly, further studies are needed to confirm these preliminary in vitro results.

## Figures and Tables

**Table 1:** Polyphenolic composition of ATLAA compared to the CTR.

Phenolic Compound	mg/g ± SD	CV% vs. Control
Gallic acid	0.032 ± 0.008 *	707.78
Procyanidin B1 + B3	0.072 ± 0.002 ***	42.97
Catechin	0.008 ± 0.001 **	512.49
Chlorogenic acid	0.521 ± 0.051 *	32.57
Procyanidin B2	0.088 ± 0.003 **	168.26
Epicatechin	0.111 ± 0.005 **	77.47
Procyanidin C1	0.025 ± 0.008 **	156.99
Rutin	0.161 ± 0.003	3.40
Quercetin-3-*O*-glucoside	0.059 ± 0.002	6.11
Esperidin	0.024 ± 0.001	−2.20
Kampherol-3-*O*-glucoside	0.026 ± 0.001	0.57
Apigenin-7-*O*-glucoside	0.006 ± 0.001 *	29.04
Kampherol-3-rhamnoside	0.003 ± 0.0001	−5.10
Naringenin	0.013 ± 0.002 ***	134.33
Phlorizin	0.383 ± 0.011 ***	90.18
Quercetin	ND	ND
Total	1.483 ± 0.05 **	49.51

Statistical significance is calculated by Student’s *t*-test analysis: * *p* < 0.05 mg/g of specific polyphenilc compouds calculated in ATLAA (Acid Treated Lyophilized Annurca Apple) vs. CTR (control); ** *p* < 0.01 mg/g of specific polyphenilc compouds in ATLAA vs. CTR; *** *p* < 0.0001 mg/g of specific polyphenilc compouds in ATLAA vs. CTR. Not detected (ND), Standard deviation (SD), CV% ( coefficient of variation %).

**Table 2:** Polyphenolic composition of ATLAA polyphenolic bioaccessible fraction vs. polyphenolic bioaccessible fraction of LAA.

Phenolic Compound	mg/g of ATLAA ± SD	CV% vs. LAA
Gallic acid	0.037 ± 0.001 ***	38.19
Procyanidin B1 + B3	0.050 ± 0.007 **	52.63
Catechin	0.012 ± 0.0003 **	16.99
Chlorogenic acid	0.155 ± 0.006	−7.75
Procyanidin B2	0.017 ± 0.003 *	36.91
Epicatechin	ND	ND
Procyanidin C1	0.036 ± 0.008	18.42
Rutin	0.065 ± 0.003 *	9.44
Quercetin-3-*O*-glucoside	0.019 ± 0.001	8.33
Esperidin	0.0013 ± 0.001 **	128.29
Kampherol-3-*O*-glucoside	0.014 ± 0.001 **	51.38
Apigenin-7-*O*-glucoside	0.006 ± 0.001 *	29.91
Narigenin	0.008 ± 0.001 **	96.45
Kampherol-3-rhamnoside	ND	ND
Phlorizin	0.250 ± 0.021 **	24.28
Quercetin	ND	ND
Total	0.684 ± 0.02 **	17.36

Statistical significance is calculated by Student’s *t*-test analysis: * *p* < 0.05 mg/g of specific polyphenilc compouds calculated in ATLAA (Acid Treated Lyophilized Annurca Apple) vs. LAA (Lyophilized Annurca Apple); ** *p* < 0.01 mg/g of specific polyphenilc compouds in ATLAA vs. control; *** *p* < 0.0001 mg/g of specific polyphenilc compouds in ATLAA vs. LAA. ND (not detected), Standard deviation (SD), CV% (coefficient of variation %).

**Table 3:** Intra-day and inter-day precision of the representative compounds of Annurca apple polyphenolic fraction.

Compound	Concentration (mg/mL)	Intra-Day Precision (%CV, *n* = 3)	Inter-Day Precision (%CV, *n* = 3)
Gallic acid	0.01	0.896	0.547
	0.05	1.682	1.300
	0.1	2.620	3.172
Procyanidin B3	0.01	2.109	6.177
	0.05	2.054	4.933
	0.1	3.027	1.114
Catechin	0.01	8.860	7.974
	0.05	1.463	1.867
	0.1	0.611	0.122
Chlorogenic acid	0.01	2.952	4.876
	0.05	2.285	2.931
	0.1	0.657	1.491
Procyanidin B2	0.01	4.049	10.747
	0.05	2.667	7.420
	0.1	1.587	6.484
Epicatechin	0.01	7.613	7.319
	0.05	7.913	1.800
	0.1	2.350	2.296
Procyanidin C1	0.01	2.185	7.715
	0.05	0.387	10.393
	0.1	3.957	4.701
Rutin	0.01	1.922	2.920
	0.05	0.126	2.913
	0.1	1.354	1.378
Phloridzin	0.01	2.339	1.868
	0.05	0.530	1.032
	0.1	0.165	1.242
Quercetin	0.01	2.276	4.260
	0.05	1.375	6.975
	0.1	0.360	4.637

CV% (coefficient of variation %).

**Table 4:** Intra-day and inter-day accuracy of the representative compounds of Annurca apple polyphenolic fraction.

Compound	Concentration (mg/mL)	Intra-Day Accuracy (% bias, *n* = 3)	Inter-Day Accuracy (% bias, *n* = 3)
Gallic acid	0.01	0.368	0.394
	0.05	1.272	1.506
	0.1	3.508	3.926
Procyanidin B3	0.01	0.377	0.292
	0.05	1.079	0.947
	0.1	2.737	2.797
Catechin	0.01	−0.564	−0.509
	0.05	0.076	−0.080
	0.1	1.312	1.284
Chlorogenic acid	0.01	−0.184	−0.196
	0.05	−1.483	−1.574
	0.1	−2.960	−2.980
Procyanidin B2	0.01	0.450	0.337
	0.05	1.578	1.152
	0.1	3.105	2.504
Epicatechin	0.01	−0.011	0.144
	0.05	0.620	1.250
	0.1	0.894	0.882
Procyanidin C1	0.01	4.326	4.011
	0.05	2.858	2.160
	0.1	4.235	3.988
Rutin	0.01	0.034	0.020
	0.05	0.100	0.001
	0.1	0.601	0.643
Phloridzin	0.01	1.883	1.921
	0.05	11.190	11.428
	0.1	19.048	19.408
Quercetin	0.01	−0.261	−0.265
	0.05	−1.561	−1.709
	0.1	−2.765	−2.788

**Table 5:** Linearity and sensitivity of the HPLC-DAD method.

Poliphenolic Standards	Linearity	Correlation Coefficient (*r*^2^)	LOQ (mg/mL)	LOD (mg/mL)	Monitoring Channel
Gallic Acid	Y = 2 × 10^7^ × −28,492	0.999	0.001	0.0005	280 nm
Procyanidin B3	Y = 2 × 10^7^ × −24,093	0.999	0.0025	0.001	280 nm
Catechin	Y = 9 × 10^6^ × −11,907	0.999	0.005	0.001	280 nm
Chlorogenic Acid	Y = 3 × 10^7^ × −30,148	0.999	0.0020	0.001	280 nm
Procyanidin B2	Y = 1 × 10^7^ × −35,836	0.999	0.0025	0.0020	280 nm
Epicatechin	Y = 1 × 10^7^ × −4563.5	0.999	0.0025	0.0020	280 nm
Procyanidin C1	Y = 5 × 10^6^ × −14,717	0.999	0.005	0.0025	280 nm
Rutin	Y = 2 × 10^7^ × −1076.9	0.999	0.001	0.0005	360 nm
Phloridzin	Y = 1 × 10^7^ × +8099	0.998	0.002	0.001	280 nm
Quercetin	Y = 5 × 10^7^ × −41,742	0.999	0.0005	0.0001	360 nm

LOQ (limit of quantification), LOD (limit of detection).

## Data Availability

The data used to support the findings of this study are included within the article.

## Supplementary Materials

The following are available online at https://www.mdpi.com/article/10.3390/foods11101453/s1, Figure S1: Chromatograms of ATLAA (Acid Treated Lyophilized Annurca Apple) recorded at 280 nm (A) and at 360 nm (B); Figure S2: Chromatograms of analytical standards recorded at 280 nm (A and B) and at 360 nm (C); Table S1: HPLC-DAD polyphenolic characterization of ATLAA.
