# Supplementary material for: Application of a Rapid and Simple Technological Process to Increase Levels and Bioccessibility of Free Phenolic Compounds in Annurca Apple Nutraceutical Product
Source: Foods. 2022 May 17;11(10):1453. doi: 10.3390/foods11101453 (PMC9141411; doi:10.3390/foods11101453)

Table S1. HPLC-DAD polyphenolic characterization of ATLAA.

No Peak	Monitoring Channel	Phenolic compound
1	280 nm	Gallic Acid
2	280 nm	Procyanidin B1+B3
3	280 nm	Catechin
4	280nm	Chlorogenic Acid
5	280 nm	Procyanidin B2
6	280 nm	Epicatechin
7	280 nm	Procyanidin C1
8	360 nm	Esperidin
9	360 nm	Rutin
10	280 nm	Quercetin-3-O-glucoside
11	360 nm	Kampherol-3-O-glucoside
12	360 nm	Apigenin-7-O-glucoside
13	360 nm	Kampherol-3-rhamnoside
14	280 nm	Phloridzin
15	280 nm	Narigenin
16	360 nm	Quercetin

Figure S1. Chromatograms of ATLAA recorded at 280 nm (A) and at 360 nm (B)

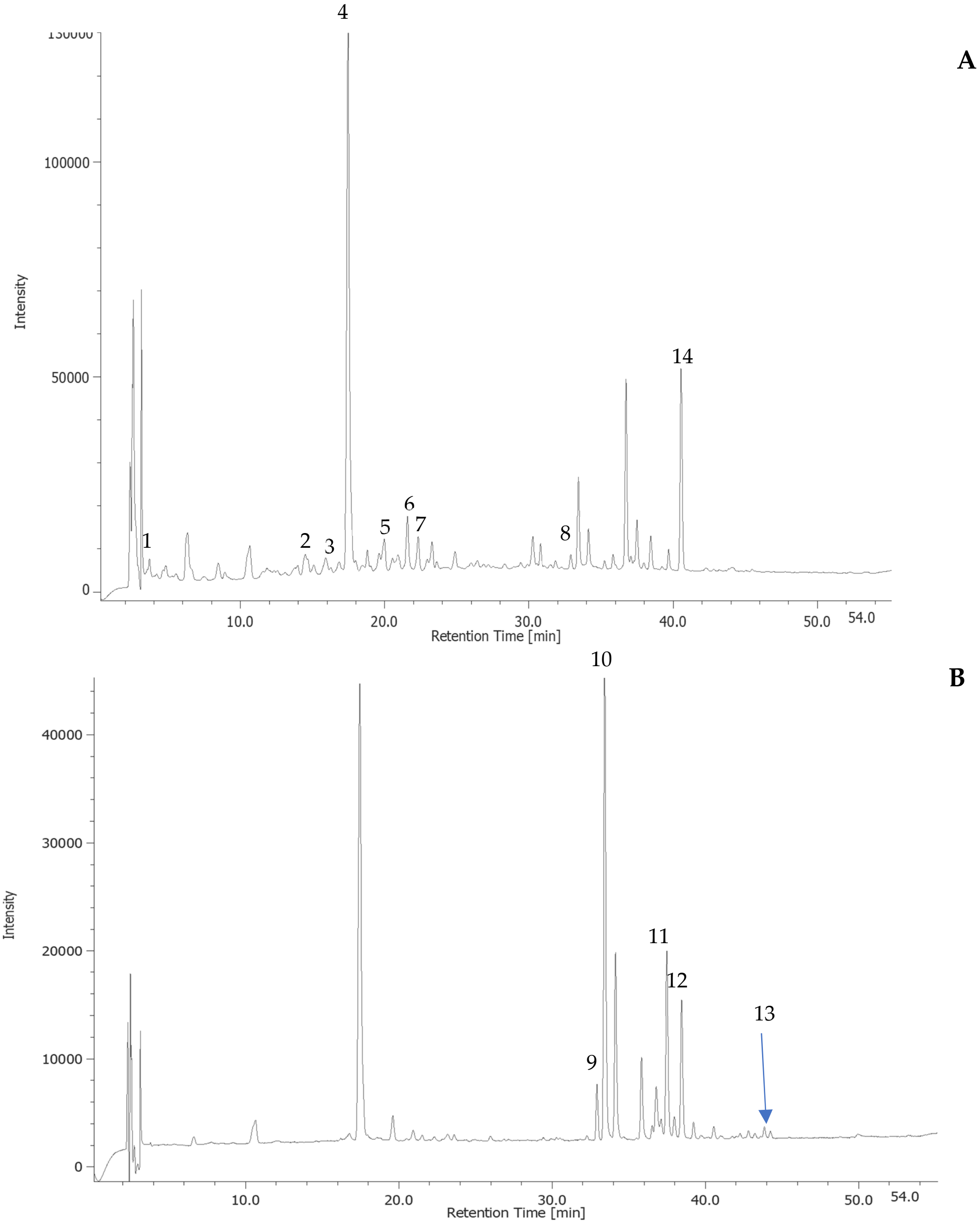

Figure S2. Chromatograms of analytical standards recorded at 280 nm (A and B) and at 360 nm (C)

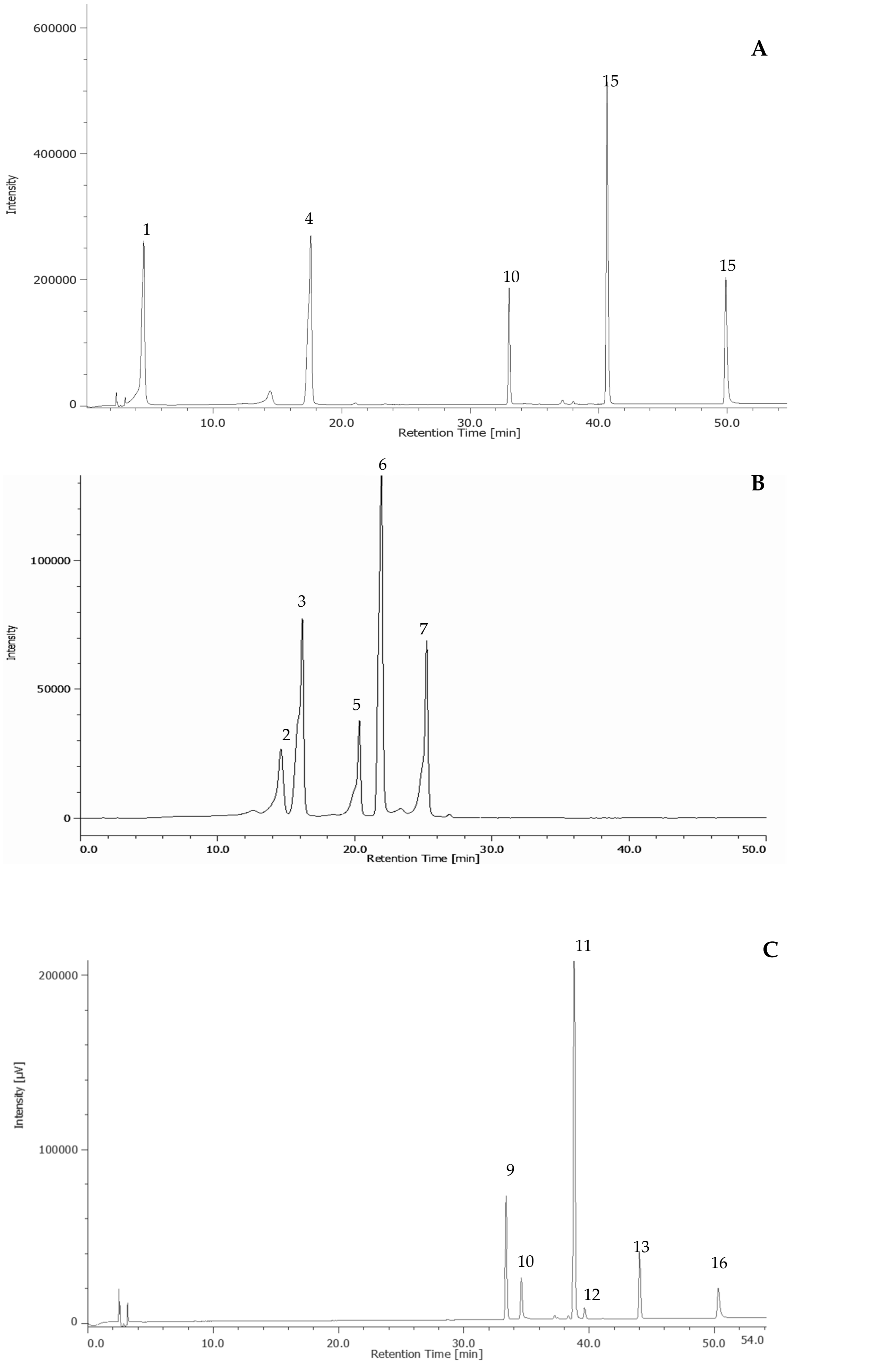

Supplement: Supplementary file 1 [file foods-11-01453-s001.zip › foods-1658701-supplementary.pdf]
